# Supplementary material for: Accounting for albedo in carbon market protocols
Source: Nat Commun. 2025 Oct 6;16:8810. doi: 10.1038/s41467-025-64317-x (PMC12500933; doi:10.1038/s41467-025-64317-x)
Supplement: Supplementary file 1 — Supplementary Information [file 41467_2025_64317_MOESM1_ESM.pdf]

Supplementary Information to "Accounting for Albedo in Carbon Market Protocols"

Supplementary Table 1. *Summary of albedo deductions and benefits by biomes. The minimum, maximum, and median project albedo deductions/benefits refer to the project within each biome that was found to have the minimum, maximum, and median project albedo deduction/benefit. We excluded four biomes with 1 or no projects located within them. Biomes are sourced from Dinerstein et al, 2017[1]. Negative values for minimum, maximum, and median indicate albedo benefits, and positive values for minimum, maximum, and median indicate albedo deductions.*

Biome	Project Count	Project Hectares	Projected Credits (million metric tons of CO_2)	Project Albedo Deduction (+)/ Benefit (-)		
				Minimum	Maximum	Median
Deserts & Xeric Shrublands	10	451,993	15.5	-13.8%	10,000% [a]	76.0%
Mangroves	8	159,130	0.5	-13.8%	62.0%	3.1%
Mediterranean Forests, Woodlands & Scrub	5	19,971	4.0	-100.9%	8,285.8%	48.0%
Montane Grasslands & Shrublands	21	449,288	136.5	-16.1%	10,000% [a]	38.9%
Temperate Broadleaf & Mixed Forests	18	1,192,567	164.0	2.9%	304.0%	27.8%
Temperate Conifer Forests	6	7,887	5.5	17.7%	10,000% [a]	165.0%
Tropical & Subtropical Coniferous Forests	4	14,989	0.3	2.9%	16.9%	6.9%
Tropical & Subtropical Dry Broadleaf Forests	22	11,629,253	15.9	-11.6%	10,000% [a]	17.7%
Tropical & Subtropical Grasslands,	62	6,353,644	107.2	-11.4%	10,000% [a]	20.8%

Savannas & Shrublands						
Tropical & Subtropical Moist Broadleaf Forests	101	29,949,181	346.2	-24.0%	10,000% [a]	11.3%

[a] The Hasler et al, 2024 dataset truncates high albedo offsets at 10,000% to avoid +/- infinity[2].

Supplementary Table 2. *Voluntary Carbon Market standards and protocols assessed with summaries of the total projected credits (in million metrics tons), minimum project albedo median, median project albedo median, and maximum project albedo median across all projects using each respective protocol. The minimum, maximum, and median project albedo deductions/benefits refer to the project from each protocol that was found to have the minimum, maximum, and median project albedo deduction/benefit.*

VCM Registry	Protocols	Project Count	Projected Credits (million metric tons CO_2)	Project Albedo Deduction/ Benefit		
				Minimum	Maximum	Median
American Carbon Registry (ACR) (https://acrcarbon.org/registry/)	AR-ACM0001	1	0.4	23.5%	23.5%	23.5%
Climate Action Reserve (CAR) (https://www.climateactionreserve.org/)	ARB Compliance Offset Protocol - U.S. Forest Projects	1	0.0	26.1%	26.1%	26.1%
Ecoregistry (ER) (https://www.ecoregistry.io/projects-list/cercarbono-co2)	AR-ACM0003	12	17.3	0.8%	33.0%	9.9%
	CERCARBONO	1	1	7.0%	7.0%	7.0%
Gold Standard (GS) (https://registry.goldstandard.org/projects)	AR-AM0003	1	0.8	11.3%	11.3%	11.3%
	AR-AM0004	1	1.8	383.6%	383.6%	383.6%
	Gold Standard: Afforestation/Reforestation	8	4.6	-23.9%	8,285.8%	17.9%

Verified Carbon Standard (VCS) (https://registry.verra.org/app/search/VCS)	AR-ACM0001	17	34.1	-20.9%	81.9%	44.7%
	AR-ACM0002	1	0.3	72.3%	72.3%	72.3%
	AR-ACM0003	72	551.5	-100.9%	10,000% [a]	18.4%
	AR-ACM0014	1	1	62.0%	62.0%	62.0%
	AR-AM0003	1	0.2	-2.3%	-2.3%	-2.3%
	AR-AM0005	2	4.8	3.7%	7.0%	5.3%
	AR-AM0014	1	1.4	20.5%	20.5%	20.5%
	AR-AMS0001	14	16.9	-1.6%	87.8%	12.3%
	AR-AMS0002	2	0.0	63.7%	192.3%	128.0%
	AR-AMS0005	2	0.2	65.5%	116.6%	91.1%
	AR-AMS0006	1	0.6	17.5%	17.5%	17.5%
	AR-AMS0007	8	14.5	-16.1%	109.6%	13.7%
	VM0007	1	62.9	15.8%	15.8%	15.8%
	VM0017	1	0.7	60.8%	60.8%	60.8%
	VM0026	1	17.7	1,548.8%	1,548.8%	1,548.8%
	VM0047	22	62.0	-11.6	84.1%	18.6%

[a] The Hasler et al, 2024 dataset truncates high albedo offsets at 10,000% to avoid +/- infinity[2].

References

1: Dinerstein, E., Olson, D., Joshi, A., Vynne, C., Burgess, N. D., Wikramanayake, E., Hahn, N., Palminteri, S., Hedao, P., Noss, R., Hansen, M., Locke, H., Ellis, E. C., Jones, B., Barber, C. V., Hayes, R., Kormos, C., Martin, V., Crist, E., Sechrest, W., Price, L., Baillie, J. E. M., Weeden, D., Suckling, K., Davis, C., Sizer, N., Moore, R., Thau, D., Birch, T., Potapov, P., Turubanova, S., Tyukavina, A., de Souza, N., Pintea, L., Brito, J. C., Llewellyn, O. A., Miller, A. G., Patzelt, A., Ghazanfar, S. A.,

Timberlake, J., Klöser, H., Shennan-Farpón, Y., Kindt, R., Jens-Peter Barnekow Lillesø, J.-P. B., van Breugel, P., Graudal, L., Voge, M., Al-Shammari, K. F., Saleem, M. An ecoregion-based approach to protecting half the terrestrial realm. *BioScience* **67(6)**, 534–545 (2017).

2: Hasler, N., Williams, C. A., Denney, V. C., Ellis, P. W., Shrestha, S., Terasaki Hart, D. E., Wolff, N. H., Yeo, S., Crowther, T. W., Werden, L. K., Cook-Patton, S. C. Accounting for albedo change to identify climate-positive tree cover restoration. *Nature Communications* **15**, 2275 (2024).
