# Supplementary material for: Accounting for albedo in carbon market protocols
Source: Nat Commun. 2025 Oct 6;16:8810. doi: 10.1038/s41467-025-64317-x (PMC12500933; doi:10.1038/s41467-025-64317-x)
Supplement: Supplementary file 2 — Reporting Summary [file 41467_2025_64317_MOESM2_ESM.pdf]

nature portfolio

Corresponding author(s): Lynn M. Riley

Last updated by author(s): 08-22-2025

Reporting Summary

Nature Portfolio wishes to improve the reproducibility of the work that we publish. This form provides structure for consistency and transparency in reporting. For further information on Nature Portfolio policies, see our Editorial Policies and the Editorial Policy Checklist.

Statistics

For all statistical analyses, confirm that the following items are present in the figure legend, table legend, main text, or Methods section.

n/a	Confirmed	
☐	☒	The exact sample size (*n*) for each experimental group/condition, given as a discrete number and unit of measurement
☐	☒	A statement on whether measurements were taken from distinct samples or whether the same sample was measured repeatedly
☒	☐	The statistical test(s) used AND whether they are one- or two-sided<br>*Only common tests should be described solely by name; describe more complex techniques in the Methods section.*
☒	☐	A description of all covariates tested
☐	☒	A description of any assumptions or corrections, such as tests of normality and adjustment for multiple comparisons
☒	☐	A full description of the statistical parameters including central tendency (e.g. means) or other basic estimates (e.g. regression coefficient) AND variation (e.g. standard deviation) or associated estimates of uncertainty (e.g. confidence intervals)
☒	☐	For null hypothesis testing, the test statistic (e.g. *F*, *t*, *r*) with confidence intervals, effect sizes, degrees of freedom and *P* value noted<br>*Give P values as exact values whenever suitable.*
☒	☐	For Bayesian analysis, information on the choice of priors and Markov chain Monte Carlo settings
☒	☐	For hierarchical and complex designs, identification of the appropriate level for tests and full reporting of outcomes
☒	☐	Estimates of effect sizes (e.g. Cohen's *d*, Pearson's *r*), indicating how they were calculated

Our web collection on statistics for biologists contains articles on many of the points above.

Software and code

Policy information about availability of computer code

Data collection	Our analyses were based on publicly available datasets as of June 2024, which we downloaded directly from Zenodo, the Harvard Dataverse, and Verra's registry of Afforestation, Reforestation, and Revegetation projects. The data and associated R scripts generated in this study have been deposited in the Zenodo database and can be accessed at https://doi.org/10.5281/zenodo.16749322.
Data analysis	All spatial analyses (spatial overlays and zonal statistics of albedo data on the project boundaries) were conducted using the `terra` package of Program R. Figures were produced using `ggplot2`. The data and associated R scripts generated in this study have been deposited in the Zenodo database and can be accessed at https://doi.org/10.5281/zenodo.16749322.

For manuscripts utilizing custom algorithms or software that are central to the research but not yet described in published literature, software must be made available to editors and reviewers. We strongly encourage code deposition in a community repository (e.g. GitHub). See the Nature Portfolio guidelines for submitting code & software for further information.

April 2023

Data

Policy information about availability of data

All manuscripts must include a data availability statement. This statement should provide the following information, where applicable:

- Accession codes, unique identifiers, or web links for publicly available datasets
- A description of any restrictions on data availability
- For clinical datasets or third party data, please ensure that the statement adheres to our policy

All input data are publicly available. Project descriptions were sourced from the registries listed in Supplementary Table 2. Project boundaries sourced from Karnik et al, 2025 were retrieved from https://zenodo.org/records/11459391. Project boundaries sourced from projects using VM0047 were sourced from .kml files on the Verra Registry at https://registry.verra.org/app/search/VCS. The albedo deduction/benefit raster layer used to calculate project median albedo was sourced from Hasler et al, 2024, at https://dataverse.harvard.edu/api/access/datafile/8550241. The data and associated R scripts generated in this study have been deposited in the Zenodo database and can be accessed at https://doi.org/10.5281/zenodo.16749322.

Research involving human participants, their data, or biological material

Policy information about studies with human participants or human data. See also policy information about sex, gender (identity/presentation), and sexual orientation and race, ethnicity and racism.

Reporting on sex and gender	This research does not involve human participants, their data, or biological material.
Reporting on race, ethnicity, or other socially relevant groupings	This research does not involve human participants, their data, or biological material.
Population characteristics	This research does not involve human participants, their data, or biological material.
Recruitment	This research does not involve human participants, their data, or biological material.
Ethics oversight	This research does not involve human participants, their data, or biological material.

Note that full information on the approval of the study protocol must also be provided in the manuscript.

Field-specific reporting

Please select the one below that is the best fit for your research. If you are not sure, read the appropriate sections before making your selection.

☐ Life sciences ☐ Behavioural & social sciences ☒ Ecological, evolutionary & environmental sciences

For a reference copy of the document with all sections, see nature.com/documents/nr-reporting-summary-flat.pdf

Ecological, evolutionary & environmental sciences study design

All studies must disclose on these points even when the disclosure is negative.

Study description	This study used the full population of available Afforestation, Reforestation, and Revegetation (ARR) projects within the Voluntary Carbon Market (VCM) with necessary geospatial data and project crediting data available, and assessed them against recently published data on albedo change due to forest restoration activities, resulting in summary statistics on the potential effect within existing VCM projects if albedo change was considered in project accounting.
Research sample	The full population of ARR projects within the VCM with accessible geospatial and project crediting data were used in this study based on those included in the open-access database of nature-based carbon offset project boundaries published in Karnik et al, 2025. All projects with a “Project Type” of “ARR” (Afforestation, Reforestation, and Revegetation) were selected for a total of 190 projects. Then, 24 that only provided geospatial data in point format—rather than polygons—were filtered out, bringing the total to 166 projects for this study. Of the 166 projects remaining, 16 projects were dropped from the assessment where they could not produce a quantified median albedo deduction or where ex-ante projected credits could not be found within corresponding project documentation available publicly on the carbon registries on which these projects are listed (Supplementary Table 2). This brought the total projects for assessment to 150. An additional 22 projects were added to those 150 projects to include projects using Verra Methodology 0047 (VM0047), which was published in late 2023 and had not yet been added to the Karnik et al, 2025 project database. All VM0047 projects on the Verra Registry as of June 2025 were used. This was done in anticipation of VM0047 becoming increasingly used within the VCM as it replaces other protocols due to its advancement of the widely-recognized dynamic baseline approach to enhancing causal attribution and additionality of a project intervention relative to traditional baseline approaches. This brought the total projects assessed to 172. We sourced geospatial boundaries for the VM0047 projects from the Verra Registry. These were converted from .kml files to polygon files for the assessment using ArcGIS Pro v3.3.0.
Sampling strategy	Sampling was not performed, as the entire accessible population was utilized in this study.

Data collection	All data are publicly available. The authors utilized VCM registries (linked in Supplementary Table 2) to search and find project documentation for each project assessed. Project boundaries sourced from Karnik et al, 2025 were retrieved from https://zenodo.org/records/11459391. Project boundaries sourced from projects using the VM0047 protocol were sourced from .kml files on the Verra Registry at https://registry.verra.org/app/search/VCS. The albedo deduction/benefit raster layer used to calculate project median albedo was sourced from Hasler et al, 2024, at https://dataverse.harvard.edu/api/access/datafile/8550241.
Timing and spatial scale	The data were downloaded in June 2024 and checked for updates in April 2025. All projects globally were utilized where data was accessible, and project size ranged from a few hectares to thousands.
Data exclusions	24 projects that only provided geospatial data in point format—rather than polygons—were excluded from the analysis. Another 16 projects were excluded where they could not produce a quantified median albedo deduction or where ex-ante projected credits could not be found within corresponding project documentation available publicly on the carbon registries on which these projects are listed. These elements (accessible polygon data, quantified albedo deductions, and publicly available credit projections) were pre-established as required for our calculations.
Reproducibility	A random 10% of projects were selected to be re-calculated as a check for reproducibility, with all steps from data collection, to GIS processing, to calculations re-produced. One project was updated based on a user error identified in these checks between the pre-print version and the accepted version of the paper. This updated project did not change the reported results. All other checks were successfully reproduced.
Randomization	The full population of projects with accessible data as described above were utilized, and therefore randomization was not utilized. In reporting results, we also grouped projects by biome and protocol (Figure 3).
Blinding	Blinding is not relevant, as no treatments were assigned to any groups within this assessment.

Did the study involve field work? ☐ Yes ☒ No

Reporting for specific materials, systems and methods

We require information from authors about some types of materials, experimental systems and methods used in many studies. Here, indicate whether each material, system or method listed is relevant to your study. If you are not sure if a list item applies to your research, read the appropriate section before selecting a response.

Materials & experimental systems

n/a	Involved in the study	
☒	☐	Antibodies
☒	☐	Eukaryotic cell lines
☒	☐	Palaeontology and archaeology
☒	☐	Animals and other organisms
☒	☐	Clinical data
☒	☐	Dual use research of concern
☒	☐	Plants

Methods

n/a	Involved in the study	
☒	☐	ChIP-seq
☒	☐	Flow cytometry
☒	☐	MRI-based neuroimaging

Plants

Seed stocks	No seed stock was used in this research.
Novel plant genotypes	No novel plant genotypes were produced in this research.
Authentication	No seed stock or novel plant genotypes were used or produced in this research.
